# Impact of dopamine-related genetic variants on physical activity in old age – a cohort study

**DOI:** 10.1186/s12966-020-00971-2

**Published:** 2020-05-24

**Authors:** Ing-Mari Dohrn, Goran Papenberg, Elisabeth Winkler, Anna-Karin Welmer

**Affiliations:** 1 Aging Research Center, Department of Neurobiology, Care Sciences and Society (NVS), Karolinska Institutet and Stockholm University, Tomtebodavägen 18 A, Solna, SE-171 65 Sweden; 2 School of Population Health, University of Queensland, Brisbane, Australia; 3 School of Public Health, University of Queensland, Herston, Australia; 4 Division of Physiotherapy, Department of Neurobiology, Care Sciences and Society (NVS), Karolinska Institutet, Stockholm, Sweden; 5 Allied Health Professionals, Function Area Occupational Therapy & Physiotherapy, Karolinska University Hospital, Stockholm, Sweden; 6 Stockholm Gerontology Research Center, Stockholm, Sweden

**Keywords:** Accelerometery, Aging, Dopamine, Genes, Physical activity, Sedentary behaviour

## Abstract

**Objectives:**

The beneficial effects of a physically active lifestyle in aging are well documented. Understanding the factors of importance for physical activity in older adults are therefore essential. Informed by animal and human data linking the dopamine system to motivation and reward processes, we investigated the associations between variations in dopamine genes and objectively measured physical activity and sedentary behaviour. Further, we aimed to verify whether higher age may exacerbate the impact of dopamine genes on physical activity.

**Methods:**

We analyzed data from 504 older adults, 66–87 years, from the population-based Swedish National study on Aging and Care in Kungsholmen (SNAC-K). Physical activity was measured with activPAL accelerometers and DNA was extracted from blood samples for genotyping. We assessed the effects of three dopamine relevant genetic variations (*DRD1*, *DRD2,* and *DRD3*) on daily time in sedentary behavior, light-intensity physical activity and moderate-to-vigorous physical activity using analyses of covariance, adjusting for sex, age and physical function.

**Results:**

Higher dopamine receptor efficacy was related to moderate-to-vigorous physical activity, but not to light-intensity physical activity or sedentary time. *DRD1* explained 2.7% of variance in moderate-to-vigorous physical activity, with more pronounced effect in people aged ≥80 years, about 10% of explained variance.

**Conclusion:**

Stronger genetic effects in older adults are in line with the well-established nonlinear effects of dopamine signaling on performance, expected to be exacerbated with aging. Individuals over 80 years, genetically predisposed to lower dopamine receptor efficacy, engaged on average 100 min/week in moderate-to-high physical activity, below the recommended levels beneficial for healthy aging. Our findings highlight that some individuals might need extra support to maintain a physically active lifestyle.

## Background

There is clear scientific evidence of the beneficial effects of physical activity and the negative consequences of sedentary behaviour on numerous health outcomes, as well as premature death [1, 2]. Regular physical activity can help maintain independence and increase the quality of life and well-being in later life [2, 3]. Older adults are therefore recommended to spend at least 150 min per week in physical activities at moderate-to-vigorous intensity [3, 4]. However, epidemiological data show that many older adults do not reach the recommended amount of physical activity and spend on average 70% of their waking hours being sedentary [5–7].

**Fig. 1:**
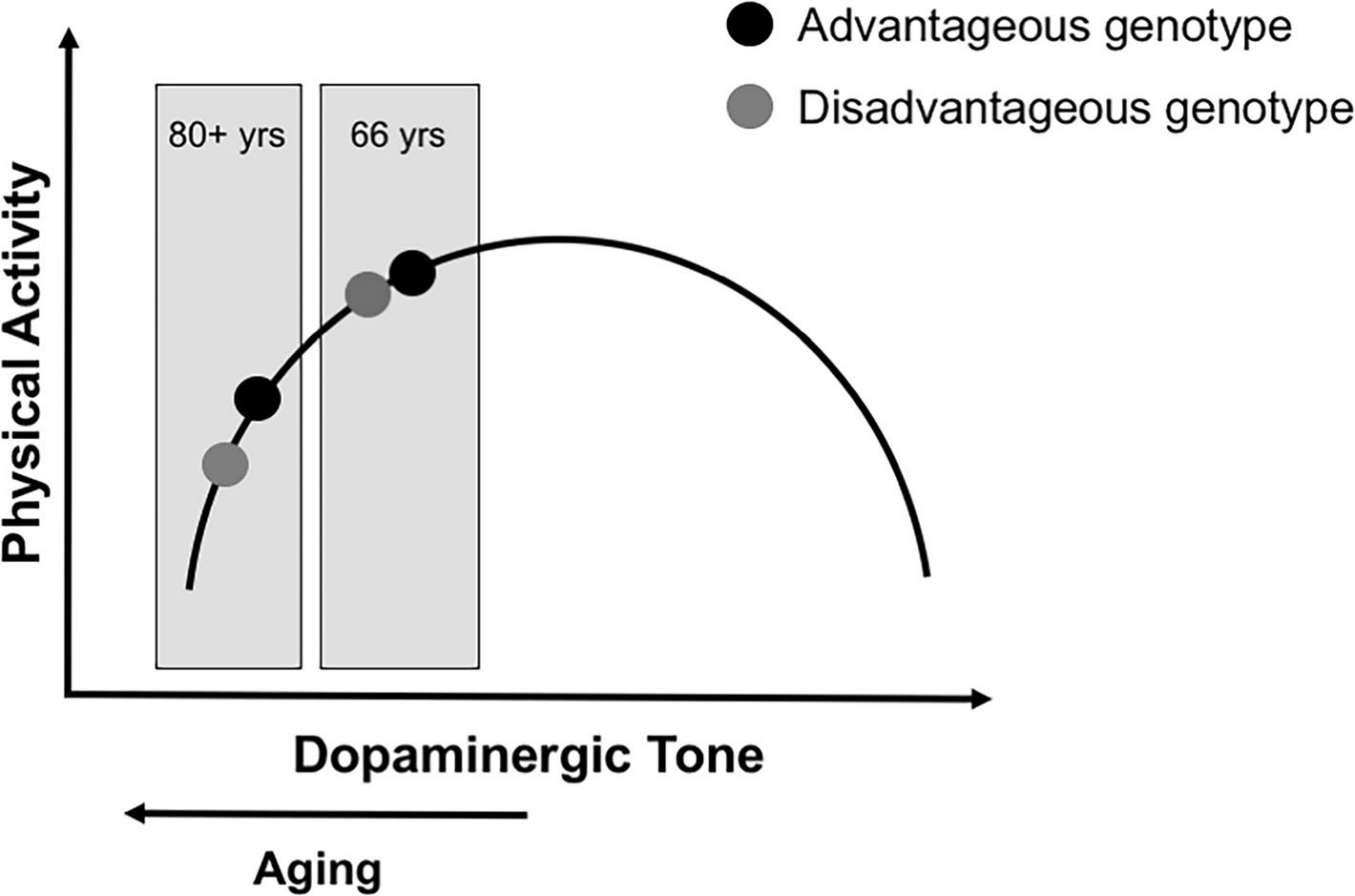
The inverted u-shaped function relating dopaminergic tone to functioning. The circles represent two individuals with different genotypes of the same gene as they experience age-related declines in the dopamine system, moving them left of the apex of the function

Understanding why people are physically active or not can contribute to planning of targeted evidence-based interventions to increase physical activity and reduce sedentary time. There are several known factors of importance for physical activity [8–10] and sedentary behaviour [11, 12] in older adults, such as social interaction, feeling of meaningfulness and joy, belief in health benefits, and exercise self-efficacy. All these factors are related to motivational and reward processes, which are associated with the dopaminergic system [13]. For example, animal data show that blocking dopamine receptors results in less engagement in voluntary exercise [14, 15]. In humans, dopamine receptor density as well as a dopamine-related genetic variation have been related to self-reported physical activity [16, 17] and measured changes in physical activity levels during intervention [18], suggesting that dopamine receptor expression might result in differences in physical activity engagement. Dopamine has also a well-established role in motor functioning [19, 20], which may influence an individual’s ability to engage in physical activity. The relationship between dopamine and cognitive performance is characterized by a well-established inverted u-shaped function [21], which likely generalizes to other domains, such as physical activity [14–16]. Aging is associated with losses in dopamine receptors and transmitter content [19], which may exacerbate the influence of dopamine-related genetic variations on physical functioning even further in a non-linear way, increasing between-person differences in performance. Our hypothesis was that higher age may exacerbate the impact of dopamine-related genetic variations on physical activity (Fig. 1).

Moreover, the current evidence on the dopamine-physical activity link is mainly based on self-reported data [16, 17, 22], which are prone to reporting bias and have limited ability to identify light-intensity physical activity, such as household chores or slow walking, and sedentary time [23, 24]. With recent technological advancements, small lightweight movement sensors (accelerometers) have become available, allowing objective assessments of physical activity [25, 26]. These devices provide a more accurate investigation of physical activity across the whole intensity spectrum.

Our aim was to use the candidate gene approach to investigate individual differences in dopaminergic modulation, focusing on three dopamine receptor-related polymorphisms (*DRD1*, *DRD2,* and *DRD3*), and their association to objective measures of physical activity and sedentary time in a population-based sample of older adults. We focus on D1 and D2-like receptor genes (i.e., D2 and D3), given that both have been involved in reward and motivational processes [27] as well as physical activity [16, 28, 29]. Moreover, by considering the effects of D2 and D3 receptors, which also function as autoreceptors [30, 31], regulating release of the neurotransmitter, we take into account the balance between pre-and postsynaptic components, which is crucial for optimal dopamine signaling [32]. Further, we aimed to verify whether advanced age may exacerbate the impact of dopamine-related genetic variations on physical activity.

## Methods

### Study population

We used data from the Swedish National study on Aging and Care in Kungsholmen (SNAC-K), consisting of persons randomly selected from eleven age groups ≥60 years living at home or in an institution in Kungsholmen, Stockholm. A total of 3363 persons (73% of all eligible people) participated in the baseline examination 2001–2004. Follow-up examinations are performed every six years for younger cohorts (aged 60–78 years) and every three years for older cohorts (≥78 years). Sampling methods and response rates are presented in detail elsewhere [27, 33]. At follow-up 2016–2018, 1287 participants from the age groups 66, 81, 84, 87, 90 and ≥ 93 years were examined and out of those 680 persons agreed to wear a movement sensor the following week (excluding persons with severe cognitive impairment or those who were not able to move indoors without assistance). In this study we included 504 participants, excluding persons with: non-valid physical activity data (*n* = 24), age ≥ 90 years (*n* = 30), no genetic data (*n* = 103), or a diagnosis of Parkinson’s disease (*n* = 19). Compared to all tested participants 2016–2018, the analytical sample included fewer women (62% vs 64%) and a larger proportion in the 66-year age group (70% vs 42%).

### Assessment of physical activity and sedentary behavior

The activPAL3 accelerometer (PAL Technologies Ltd., Glasgow), a small and slim thigh-worn movement sensor, was used for objective assessment of physical activity and sedentary time. The sensor uses information about thigh position combined with acceleration and determines body posture and movement, i.e. sitting/lying or standing and stepping speed (cadence) with high accuracy [34, 35]. Participants were asked to continue with their usual habits while wearing the sensor for seven consecutive days during all waking hours, excluding water-based activities. Written instructions and a log-sheet to record time for attachment and removal each day were provided. The device was returned by mail in a prepaid envelope.

### Data processing activPAL

The activPAL files were processed using the PALbatch software v 8.10.6.33. A custom-made syntax for SAS programming system was used for further analyses to remove non-wear time and time in bed [35, 36]. Variables used were daily time in sitting, daily time in light-intensity physical activity (time stepping at a cadence < 100 steps/min plus daily time standing), and daily time in moderate-to vigorous physical activity (time stepping at a cadence ≥100 steps/min) [34, 37, 38]. We included participants who provided at least four valid days of accelerometer data, i.e. at least 600 min of wear time per day during waking hours [35, 39].

### Genotyping

Standard methods were used to extract DNA from peripheral blood samples. Single nucleotide polymorphisms (SNPs) of dopamine D1 (*DRD1*; rs4532), D2 (*DRD2*/*ANKK1/Taq1A*; rs1800497), and D3 (*DRD3*/*Ser9Gly*; rs6280) receptor genes were examined. More specifically, SNPs were genotyped using MALDI-TOF analysis on the Sequenom MassARRAY platform at the Mutation Analysis FacilitWy, Karolinska Institutet [40]. We performed quality control at DNA-sample level, assay level, as well as the level of multiplex assay pool. The genotype frequencies of the *DRD1* polymorphism (rs4532) were 192 for T/T, 242 for C/T, and 70 for C/C. For *DRD2* (*ANKK1/ TaqIA*; rs1800497), the distributions were 329 (A2/A2), 153 (A2/A1), and 22 (A1/A1), and the corresponding distributions for *DRD3* (*Ser9Gly*, rs6280) were 208 (T/T), 248 (T/C), and 48 (C/C). While *DRD1* and *DRD2* were in Hardy–Weinberg equilibrium (*p*s > 0.1), C-homozygotes were under- and T-carriers overrepresented for *DRD3*, chi-square (1) = 4.42, *p* < 0.05 (expected frequencies: 218 (T/T), 226 (C/T), and 59 (C/C)).

Evidence suggests that the *DRD1* C allele is associated with higher D1 receptor efficiency. Young C homozygotes have superior cognitive control than carriers of the T allele [41]. The C allele is more common in persons with bipolar disorder [42] who also have increased dopamine signaling [43]. Further, the C allele is related with an increased rate of nicotine dependence [44], likely reflecting stronger reinforcing effects of nicotine due to increased dopamine signaling [45]. With respect to the *DRD2* polymorphism, also known as Taq1A, studies have shown a direct link to D2 receptor density. The A1 allele has been related to a reduced number of receptor binding sites in the brain (e.g. [46]) and worse outcomes on cognition (e.g. [47]). With respect to *DRD3*, the C-allele has been associated with higher affinity to dopamine, leading to lower dopamine signaling [48]. Results from studies with cognition also suggest that the T/T genotype is beneficial (e.g. [49]).

### Physical function

We used two tests to control for limitations in physical function that may restrict physical activity: the 5 times sit to stand test (5 STS) and one-leg stance, eyes open (OLS). The 5 STS was performed by asking the participants to stand up and sit down five times as fast as they could, without using the arms and categorized as ability to perform five consecutive chair stands (yes/no) [50] For the OLS, each leg was tested twice, and the best score was used and categorized as ability to stand ≥5 s (yes/no) [51].

### Socio-demographic and health variables

Marital status (i.e., married/living together (yes/no), and use of walking aid (yes/no) were derived from interviews. Body mass index was calculated from weight and height measured using standard methods.

### Statistical analyses

Data were analyzed using SPSS for Windows 15.0 (SPSS, Chicago, IL). For the analyses we categorized participants into two age groups: 66 years and 81–87 years. We conducted analyses of covariance (ANCOVAs) with cohort (66 vs. 81–87) and the three dopamine-related genetic variations as between-subject factors (*DRD1*, *DRD2*, and *DRD3*) and daily time spent sitting, in light-intensity physical activity, and in moderate-to-vigorous physical activity as dependent variables. All models were adjusted for age (except for models involving 66-year-old participants only), sex and physical function (5 STS and OLS). Further adjustment for waking wear time did not change the results (data not shown). First, we examined the unique impact of SNPs (*DRD1*, *DRD2*, or *DRD3),* adjusting for the four covariates (age, sex, 5-STS and OLS). However, given that the effect of one SNP may be counteracted by the effects of the other SNPs, in a second set of models, additional adjustments were made for main (*DRD1*, *DRD2*, *DRD3*) and interactive effects among these SNPs (i.e., *DRD1 × DRD2, DRD1 × DRD3, DRD2 × DRD3, and DRD1 × DRD2 × DRD3*), which were included in the analyses as factors. Again, the same four covariates were included in the analyses (age, sex, 5-STS and OLS). Such patterns are particularly likely for the selected dopamine SNPs, which code for pre- and postsynaptic receptors. D2 and D3 receptors function also as autoreceptors [30, 31], regulating release of the neurotransmitter. It has been shown that for optimal dopamine signaling, the balance between pre-and postsynaptic components is crucial [32]. Interactions were only considered reliable with sufficient sample sizes (> 10%) in each cell to avoid spurious findings. All models were performed first in the total sample, involving cohort as between-subject factor, and then stratified by age (66 years versus 81–87 years).

As common in candidate genes studies, partial eta squared was used to indicate effect sizes, which can be directly interpreted as percent of variance in physical activity explained by independent variables. The alpha level was set to a Bonferroni-corrected level of *p* = 0.006 (three (*DRD1*, *DRD2* and *DRD3*) x three (sitting, light-intensity physical activity and moderate-to-vigorous physical activity): 0.05/9 = 0.006). Cases exceeding ±3.29 standard deviations were treated as univariate outliers [52], which resulted in the exclusion of two cases for light and moderate-to-vigorous physical activity (*n* = 502), and one case for sedentary behavior (*n* = 503).

## Results

Sample characteristics by age are presented in Table 1. In Table 2, the main results from the analyses involving *DRD1* are presented for Model 1 (adjusting for age, sex and physical function) and Model 2 (additionally adjusting for main and interactive effects of SNPs). Analyses revealed only an association between *DRD1* and moderate-to-vigorous physical activity in the total sample trending toward statistically significant, *F*(2,492) = 3.004, *p* = 0.051, partial-eta squared = 0.012 (Model 1), but not for *DRD2*, *F*(2,492) = 0.023, *p* = 0.977, partial-eta squared = 0.000, and *DRD3*, *F*(2,492) = 2.093, *p* = 0.124, partial-eta squared = 0.008. Moreover, there were no interactions between cohort and *DRD1, F*(2,492) = 1.716, *p* = 0.181, partial-eta squared = 0.007*, DRD2, F*(2,492) = 0.591, *p* = 0.544, partial-eta squared = 0.002, or *DRD3, F*(2,492) = 0.099, *p* = 0.906, partial-eta squared = 0.000. Non-significant associations between SNPs and sedentary behavioral and light-intensity physical activity are presented in Table S1 in the supplementary. The association between *DRD1* and moderate-to-vigorous physical activity became even more evident in the second model, when taking into account the other two polymorphisms and their interaction effects in the analyses, *F*(2,474) = 6.531, *p* = 0.002, partial-eta squared = 0.027. More specifically, C-homozygotes were significantly more active than T-homozygotes, *t*(1, 261) = 3.28, *p* = 0.001, and heterozygotes, *t*(1,309) = 3.29, *p* = 0.001. The latter two groups did not differ from each other (Model 2 for total sample). Moreover, none of the gene-gene interactions were significant.

**Table 1:** Sample characteristics stratified by age group

	66 years (*n* = 357)	81–87 years (*n* = 147)
Female, n (%)	212 (59.4)	101 (68.7)
Married/living together, n (%)	233 (65.3)	73 (49.6)
Body mass index, M (SD)	26.0 (3.8)	25.8 (3.9)
Use walking aid, n (%)	5 (1.4)	33 (22.4)
5 times sit to stand, not able, n (%)	6 (1.7)	27 (18.4)
One-leg stance, < 5 s, n (%)	25 (7.0)	75 (51.0)
Sitting, min/day, M (SD)	506.13 (96.8)	521.1 (82.8)
Light-intensity PA, min/day, M (SD)	319.3 (93.6)	291.1 (80.1)
Moderate-to-vigorous PA, min/day, (SD)	39.6 (24.5)	20.6 (20.5)
Accelerometer wear time, min/day, M (SD)	866.6 (61.3)	832.7 (63.2)
DRD1 (T/T; C/T; C/C), n	137/171/49	55/71/21
DRD2 (A2/A2; A2/A1; A1/A1), n	229/113/15	100/40/7
DRD3 (T/T; T/C; C/C), n	151/171/35	57/77/13

*M* mean, *SD* standard deviation, *PA* physical activity, *DRD1* dopamine D1 receptor polymorphism, *DRD2* dopamine D2 receptor polymorphism; *DRD3* dopamine D3 receptor polymorphism

**Table 2:** Estimated marginal means (standard error) and *p*-values for pairwise comparisons of moderate-to-vigorous physical activity in min/day, as a function of *DRD1* genotype, in the total sample and stratified by age

	Total sample		66 years		81–87 years	
DRD1 genotype	*Model 1*	*Model 2*	*Model 1*	*Model 2*	*Model 1*	*Model 2*
TT (lower efficacy)	32.7 (26.6, 38.7)	30.5 (23.3, 37.7)	38.8 (34.7, 42.9)	36.6 (28.4, 44.9)	14.8 (10.1, 19.6)	15.2 (8.2, 22.1)
CT (intermediate efficacy)	37.7 (31.6, 43.8)	32.7 (27.9, 37.5)	39.8 (36.1, 43.5)	32.1 (24.1, 40.2)	23.1 (18.9, 27.3)	24.5 (17.7, 31.3)
CC (higher efficacy)	39.8 (32.4, 47.2)	49.9 (40.8, 59.0)	40.8 (33.9, 47.8)	52.1 (40.3, 63.8)	27.2 (19.5, 34.9)	35.8 (26.8, 44.8)
TT vs. CC (*p*-value)	0.043	0.001	0.626	0.036	0.007	0.000
TT vs. CT (*p*-value)	0.041	0.618	0.727	0.442	0.012	0.064
CT vs. CC (*p*-value)	0.538	0.001	0.798	0.007	0.354	0.049

*DRD1* = dopamine D1 receptor polymorphism

Model 1 was adjusted for age, sex and physical function (5 STS and OLS) and included cohort as a factor in the total sample. Main effects of the two other SNPs are not included in Model 1. In Model 2, additional adjustments were made for main (i.e., *DRD1*, *DRD2*, *DRD3*) and interactive effects between the other SNPs (i.e., *DRD1* x *DRD2*, *DRD1* x *DRD3*, *DRD2* x *DRD3*, and *DRD1* x *DRD2* x *DRD3*)

Stratifying the sample in relatively younger (age = 66) and older adults (age = 81–87) revealed more pronounced effect sizes in the older age cohort (Model 2). Notably, the association in relatively younger adults was not significant in the first model, *F*(1,349) = 0.135, *p* = 0.874, partial-eta squared = 0.001. However, in relatively older adults the association was marginally significant, *F*(1,140) = 5.019, *p* = 0.008, partial-eta squared = 0.067. When taking into account the other dopamine SNPs and interaction effects in the analyses, the association between *DRD1* and moderate-to-vigorous physical activity became marginally significant also in the relatively younger adults, with *DRD1* explaining 2.7% of variance in moderate-to-vigorous physical activity, *F*(1,329) = 4.553, *p* = 0.011, partial-eta squared = 0.027. The effect of *DRD1* on moderate-to-vigorous physical activity was even more pronounced in relatively older adults, *F*(1,122) = 6.886, *p* = 0.001, partial-eta squared = 0.101, accounting for about 10% of variance.

## Discussion

We investigated the associations between predispositions in dopamine-related genetic variations and physical activity and sedentary time in older adults. Our findings suggest that higher dopamine receptor efficacy (*DRD1* C-homozygotes) is related to more moderate-to-vigorous physical activity. We did not observe this to be the case for light-intensity physical activity or sedentary time. Importantly, the effects remained when adjusted for individual differences in physical functions. Further, we found that the impact of dopaminergic SNPs on moderate-to-vigorous physical activity was more pronounced among people aged 80 years and older. Thus, our findings suggest that individual differences in dopaminergic modulation may influence motivation and reward-processes relevant for engaging in more intense physical activity especially among the oldest-old.

The fact that a D1-receptor polymorphism was associated with higher levels of moderate-to-vigorous physical activity is in line with the classical view of a crucial role of D1 receptors in positive reinforcement and reward [27]. It should be noted that D2-receptor mediated mechanisms also contribute to motivational behaviour [53], for instance, though their role as autoreceptors regulating transmitter levels. However, these effects may be more difficult to reveal, given the low expression of auto- relative to post-synaptic receptors [30, 31]. A recent study showed that higher dopamine signalling supports changes in physical activity during an intervention, but not at baseline [18]. This study investigated another genetic variation in the *DRD2* gene, associated with differences in endogenous dopamine, but not receptor density. A physical exercise intervention study in older adults, measuring D2 receptor density in the reward system, documented decreased D2 receptor availability, which is likely due to increased endogenous dopamine [28]. Accordingly, increased dopamine release may be a consequence of a physical activity intervention and support motivational or reward processes though D2-related mechanisms. The stronger genetic effect in older age is in line with the inverted U-shaped function (Fig. 1), which describes the relationship between dopaminergic modulation and performance, such as cognitive [54] and physical function [55]. To the best of our knowledge, this pattern has not previously been reported for physical activity.

Small effects sizes are very common in behaviour-genetic studies, typically explaining around 1% of variance. In this study, the observed effects size estimates were particularly high in individuals over 80 years. The differences in daily moderate-to-vigorous physical activity between individuals with more advantageous genotype and those with a disadvantageous genotype were around 20 min per day, which clearly could contribute to better health and well-being [2, 3].

The finding that individuals with higher dopamine D1 receptor efficacy actively engage in more intense physical activities supports the results from a previous study, showing that self-reported intensity of physical activity was associated with higher receptor density in the dopamine system [16]. Moreover, our findings are in line with the results from den Hoed and colleagues [56], who used objective assessment of physical activity in a twin study to investigate the role of genetic factors in physical activity regulation. They found that heritability explained 47% of the variance in time spent in moderate-to-vigorous physical activity. In contrast to our results, they also found a genetic component for time spent in sedentary behaviour. Still, the lack of associations with sedentary time and genetic differences in dopamine in this study is not surprising, given that the dopamine system seems to play a bigger role in more intense physical activity. Sedentary time and light-intensity physical activity are highly correlated, and older adults who spend less time sitting do not necessary spend more time in moderate-to-vigorous physical activity, but rather in activities with light-intensity [57, 58]. Hence, the associations with sedentary behaviour found by den Hoed and colleagues [56] may be related to other genes than those involved in the dopamine system.

In addition, as noted above, exercise intervention studies have documented both increased dopamine transmitter as well as receptor availability after intervention [28, 29]. Consequently, individuals with a stronger dopaminergic tone may be more likely to engage in physical activity. Environmental exposure, such as being more physically active may, in turn, enhance expression of a particular gene via epigenetic mechanisms, thereby resulting in stronger genetic effects, which are further exacerbated with aging. Such feedback-loops are likely related to intensity, which may also be the reason why we did not see any association between dopamine SNPs and light-intensity physical activity.

An important strength of our study is that we used objective assessment of physical activity and thereby reduced the likelihood of misclassification compared to self-reports. Our study focuses on well-described candidate genes in the literature, which have been related to inter-individual differences in brain and performance measures and which, according to theory, are related to the dopaminergic reward and motivational system. However, Rosso et al. who investigated the link between a genetic variation in the *DRD1* gene and physical activity did not find any association [18]. The missing link is very likely due to fact that the investigated *DRD1* polymorphisms may not result in strong functionally relevant interindividual differences with respect to receptor efficacy, as it has not been related to other functional outcomes in previous studies. Moreover, although our sample size may be small for genetic studies aiming at discovering new gene-phenotype links, it is reasonably powered for theory-driven candidate genes studies as in the present case [18, 54]. That said, our sample is not suitable to reveal gene-gene interactions. It should also be acknowledged that other dopamine SNPs likely contribute to physical activity, but their effects may not be picked up in such small samples and may, therefore, not be useful as biomarkers for individual differences in physical activity. From our data, it is evident that the investigated genetic variations in *DRD2* and *DRD3* influenced the effects of *DRD1* on physical activity, although they were not directly associated with physical activity. Optimally, a polygenic score, considering many SNPs, should be created to reflect individual differences in dopamine signaling.

Another strength of this study is the population-based sample, but as in any study, participants may be healthier and more physically active than the general population. Due to the physical activity assessment method, we did not include participants with severe cognitive impairment or those who could not move indoors without assistance. The fact that our study sample was positively selected and healthier than older adults in general, may have attenuated the genetic effect on physical activity. Independent replication studies in other populations are needed to confirm the observed association.

## Conclusions

This study adds important knowledge for public health initiatives, since adherence to physical activity recommendations is likely more challenging for individuals who lack a biological drive to be active. We found a genetic effect among older individuals translating into moderate effect sizes, with carriers of the disadvantageous genetic variant engaging on average 100 min/week in moderate-to-high physical activity. This is clearly under the recommended level of 150 min/week, with an increased risk of unhealthy aging. Interventions aiming to increase physical activity and reduce sedentary behaviour in older adults should not only consider well-known correlates such as cognitive, social, and environmental factors, but also acknowledge the possible impact of genetic factors. Consequently, in terms of personalized physical activity prescription, some individuals might need extra support to maintain a physically active lifestyle.

## Supplementary information

**Additional file 1: Table S1.** Effects of SNPs on sedentary behavior and light-to-moderate physical activity.

## Data Availability

The datasets used during the current study are available from maria.wahlberg@ki.se, on reasonable request.

## Abbreviations

5 STS: 5 times sit to stand test
DRD1: Dopamine D1 receptor polymorphism
DRD2: Dopamine D2 receptor polymorphism
DRD3: Dopamine D3 receptor polymorphism test
OLS: One-leg stance
SNP: Single nucleotide polymorphisms
