# Supplementary material for: Impact of dopamine-related genetic variants on physical activity in old age – a cohort study
Source: Int J Behav Nutr Phys Act. 2020 May 24;17:68. doi: 10.1186/s12966-020-00971-2 (PMC7245799; doi:10.1186/s12966-020-00971-2)
Supplement: Supplementary file 1 — Additional file 1: Table S1. Effects of SNPs on sedentary behavior and light-to-moderate physical activity. [file 12966_2020_971_MOESM1_ESM.docx]

**Table S1.** Effects of SNPs on sedentary behavior and light-to-moderate physical activity.

|  | Sedentary Behavior | Light-to-moderate PA |
| --- | --- | --- |
| *DRD1* | *F*(2,493) = 0.064, *p* = 0.908,  partial-eta squared = 0.000 | *F*(2,492) =0 .262, *p* = 0.769,  partial-eta squared = 0.001 |
| *DRD2* | *F*(2,493) = 1.397, *p* = 0.248,  partial-eta squared = 0.006 | *F*(2,492) = 1.153, *p* = 0.317,  partial-eta squared =0 .005 |
| *DRD3* | *F*(2,493) = .072, *p* = 0.930,  partial-eta squared = 0.000 | *F*(2,492) = .495, *p* = 0.495,  partial-eta squared = 0.003 |
| *Cohort x DRD1* | *F*(2,493) = .876, *p* = 0.417,  partial-eta squared = 0.004 | *F*(2,492) = .415, *p* = 0.660,  partial-eta squared = 0.002 |
| *Cohort x DRD2* | *F*(2,493) = .286, *p* = 0.751,  partial-eta squared = 0.001 | *F*(2,492) = .072, *p* = 0.931,  partial-eta squared = 0.000 |
| *Cohort x DRD3* | *F*(2,493) = .215, *p* = .806,  partial-eta squared = 0.001 | *F*(2,492) = .381, *p* = 0.683,  partial-eta squared = 0.002 |

PA=physical activity, DRD1 = dopamine D1 receptor polymorphism, DRD2 = dopamine D2 receptor polymorphism; DRD3 = dopamine D3 receptor polymorphism.
